# Unraveling Omenn syndrome in a newborn: A case report

**DOI:** 10.1016/j.jdcr.2024.10.006

**Published:** 2024-10-25

**Authors:** Oussama Mghirbi, Mariam Barka, Donia Brahem, Maha Taamli, Amani Khlifi, Sonia Merchaoui, Nabiha Mahdhaoui

**Affiliations:** a Department of Resuscitation and Neonatal Medicine, Farhat Hached University Hospital, Sousse, Tunisia; b University of Sousse, Ibn El Jazzar Medical Faculty of Sousse, Sousse, Tunisia

**Keywords:** erythroderma, neonatal, Omenn syndrome, RAG1, RAG2, severe combined immunodeficiency

## Introduction

Neonatal erythroderma is a critical condition characterized by skin inflammation affecting over 90% of the body surface, which can be life-threatening in the first month of life.^1^ It requires prompt diagnosis and urgent treatment to prevent fatal outcomes. Causes include infections, metabolic disorders, ichthyosis, inflammatory diseases, and immunological deficiencies.^2^

Omenn syndrome (OS) is a rare autosomal recessive form of severe combined immunodeficiency (SCID) caused by mutations in the RAG1 or RAG2 genes. These mutations significantly reduce the function of proteins essential to the diversity of B and T lymphocyte receptors and limit their ability to fight infection. B lymphocytes are reduced in number, while T lymphocytes, although normal in quantity, are abnormal and provoke autoimmune reactions often leading to neonatal erythroderma.^3^ Recognizing its unique biological and immunological features is essential for timely treatment to save affected newborns.^4^

This study aims to review the clinical and biological characteristics of neonatal erythroderma and OS to enhance clinician awareness of this rare condition and stress the importance of early diagnosis for effective treatment.

## Case report

We report a male newborn delivered via cesarean at 40 weeks' gestation from a singleton pregnancy. He is the first child of nonconsanguineous parents. The pregnancy was uneventful with no family history of infant death, skin or genetic disorders. Birth weight was 4000 g. He received tuberculosis and hepatitis B vaccinations according to the Tunisian schedule and was discharged on exclusive breastfeeding.

**Fig 1:**
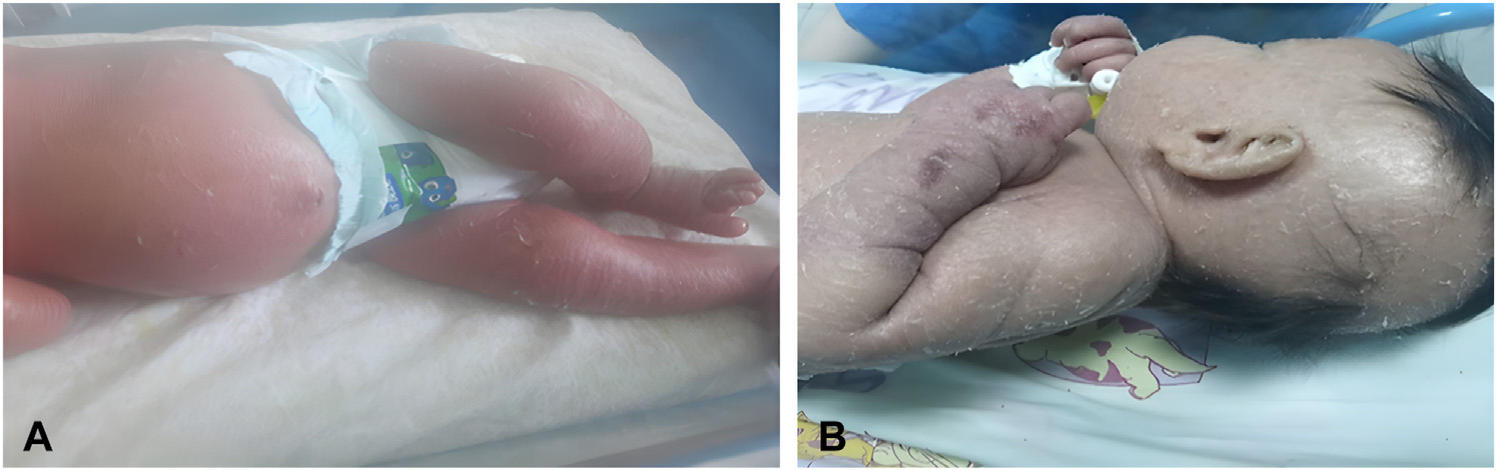
**A,** Erythroderma associated with exfoliative dermatitis. **B,** Alopecia associated with generalized exfoliative dermatitis.

At 15 days, he developed erythroderma and desquamation, initially treated with emollients without improvement. At 24 days, he presented with fever and was admitted to our department. On admission, he weighed 3850 g (150 g loss). Examination showed diffuse erythroderma, desquamation, alopecia, and absent eyelashes and eyebrows (Fig 1, *A* and *B*). Abdominal examination revealed a soft abdomen, a liver palpable 1 cm below the costal margin and a nonpalpable spleen.

Laboratory tests showed a C-reactive protein (CRP) at 68 mg/L, leukocytosis (28,800/mm³), lymphocytosis (20,448/mm³), eosinophilia (288/mm³), hemoglobin of 12 g/dL, and thrombocytopenia (88,000/mm³). Serum electrolytes were normal: sodium 141 mmol/L, urea 3 mmol/L, creatinine 23 μmol/L. Chest radiography was unremarkable with a visible thymus.

He was treated with a third-generation cephalosporin and aminoglycoside for suspected postnatal infection. Dermatologic consultation suggested congenital ichthyosis. His condition initially improved, with fever resolution and decreased CRP, but thrombocytopenia persisted.

On day 10 of admission, the patient developed recurrent fever, splenomegaly, followed by hepatomegaly and inguinal lymphadenopathy. Tests revealed anemia (hemoglobin 7 g/dL), leukocytosis (26,300/mm³), eosinophilia (2980/mm³, 11%), worsening thrombocytopenia (15,000/mm³), elevated liver enzymes (alanine aminotransferase 323 IU/L, aspartate aminotransferase 110 IU/L), high CRP (125 mg/L), impaired renal function (urea 16 mmol/L, creatinine 86 μmol/L) and hyponatremic dehydration.

Blood cultures were positive for Pseudomonas aeruginosa and Acinetobacter baumannii. Urine culture revealed Candida albicans, while viral serologies were negative. Treatment consisted of broad-spectrum antibiotherapy (Imipenem, Colistin, Amikacin) and anti-fungal therapy (Fluconazole) and correction of hematological and hydroelectrolytic disorders.

Immune evaluation confirmed SCID consistent with OS, characterized by low naive CD4 T-cell count (300 cells/mm³), absence of B cells, 40% eosinophilia, hypogammaglobulinemia (0.3 g/L), and elevated IgE (2140 IU/L).

Despite interventions, his condition worsened, leading to death from refractory septic shock before hematopoietic stem cell transplantation (HSCT).

Genetic testing identified a homozygous deleterious variant in the RAG1 gene: NM_00448.3: c.519delT (p.Glu174SerfsTer27). Both parents were heterozygous carriers of this variant.

## Discussion

Neonatal erythroderma requires quick recognition as it may indicate a life-threatening condition.^1^ OS, a rare autosomal recessive form of SCID, typically presents in the first months of life.^5^ Early diagnosis is crucial since HSCT is the sole curative option.^6^ Key clinical features include exfoliative dermatitis, erythroderma, alopecia, lymphadenopathy, hepatosplenomegaly, recurrent infections, and failure to thrive.

Aleman et al described OS as a triad of erythroderma, hepatosplenomegaly, and lymphadenopathy, present in 80% of cases.^7^ Additional signs, such as growth retardation, and severe recurrent infections, warrant an immune evaluation for OS. Notably, the signs of OS can evolve over time, which was evident in our observation where clinical manifestations appeared sequentially, often leading to misdiagnosis.^8^ The newborn in our observation initially presented only with cutaneous involvement from the age of 15 days, and it was only after 20 days that hepatosplenomegaly appeared, after which the clinical signs accelerated, most likely precipitated by infections.

In cases of erythroderma accompanied by fever, infectious diseases such as Staphylococcal scalded skin syndrome and congenital cutaneous candidiasis must be considered.^1^^,^^9^ Other differential diagnoses include Netherton syndrome, Atopic dermatitis, graft-versus-host disease, congenital ichthyosis, and various immunodeficiency syndromes.^5^^,^^10^ Table I outlines the clinical and biological characteristics that aid in the differential diagnosis of OS.

**Table I:** Differential diagnosis of Omenn syndrome^1^^,^^5^^,^^9^^,^^10^

Disorder	Suggestive findings
Omenn syndrome	• Erythroderma, hepatosplenomegaly, lymphadenopathy, alopecia, failure to thrive, recurrent infections. • Increased serum Ig E, eosinophilia. • Histological features: epidermal hyperplasia, acanthosis, parakeratosis inflammation, both dermal and epidermal, dermal lymphocytic T cells infiltrate, lymphocyte/macrophage ratio > (50:50). • Very similar to Graft-versus-host disease. • Genetic: Mutation gene RAG 1 et RAG 2.
Graft-versus-host disease	• Alopecia. • Severe failure to thrive. • Recurrent atypical infections. • Histological features: very similar to Omenn syndrome, epidermal thickness.
Atopic dermatitis	• Red, scaly, crusted lesions, dry skin. • Acute lesions may include vesicles on extensor surfaces. cheeks or scalp. • Sparing the diaper area. • Increased serum IgE levels, eosinophilia. • Histological features: epidermal hyperplasia, spongiosis, and perivascular lymphocytic infiltrate.
Netherton's syndrome	• Ichthyosiform erythroderma • Hair is usually sparse and grows slowly. • Dehydration, hypothermia. • Hypernatremia, normal lymphocyte count. • Histological features: psoriasiform hyperplasia, skin inflammation, thinning, diminished granular layer, dyskeratosis, infiltrate including neutrophils and/or eosinophils, and vascular dilatation, bamboo hairs. • Genetic: SPINK 5 mutation.
Infectious diseases	• Staphylococcal scalding syndrome: infants present with fever, irritability, and a macular rash that evolves into diffuse erythema with superficial desquamation that progress rapidly. • Congenital cutaneous candidiasis: presents at birth or in the first few days of life as small diffuse, erythematous macules and pustules often affecting the palms of the hands and soles of the feet Preterm infants may show erythroderma due to immature epidermal keratinization.

In our case, the missing skin biopsy could have aided in orienting the diagnosis (Table I). It should have been performed if the dermatological lesions did not improve with emollients or if cutaneous ichthyosis was suspected.

OS is distinguished from other SCIDs by enlarged lymphoid tissue, severe erythroderma, and its autoimmune and atopic features, such as constant hypereosinophilia, absent of immunoglobulin A and M and elevated immunoglobulin E levels, reflecting immune dysregulation.^4^^,^^8^ Like clinical and biological signs of OS may appear later in the course of the disease.

OS is a genetically heterogeneous disease, most commonly caused by mutations in RAG-1 and RAG-2 genes.^8^ These mutations result in oligoclonal proliferation of autoreactive T cells and a marked reduction in circulating B cells. Abnormal T cells infiltrate organs and release a variety of cytokines that promote autoimmune and allergic inflammation.^8^ Without treatment, combining HSCT with immunosuppressive drugs, the prognosis is fatal.^4^

## Conclusion

This case underscores the need to consider OS in the differential diagnosis of persistent neonatal erythroderma. Prompt recognition of clinical and biological signs pointing to OS and rapidly performing immunological and genetic evaluations, are essential for early diagnosis and timely HSCT, the only curative treatment.

**Key points:**

• Persistent neonatal erythroderma may indicate severe combined immunodeficiency.
• Erythroderma, hepatosplenomegaly, and lymphadenopathy are suggestive of severe combined immunodeficiency, although these signs may appear gradually.
• Early genetic analysis is crucial for diagnosis and treatment.
• Prognosis is poor without early treatment and HSCT.

## Conflicts of interest

None disclosed.
